# Study on Seismic Behavior of Earthquake-Damaged Joints Retrofitted with CFRP in Hybrid Reinforced Concrete–Steel Frames

**DOI:** 10.3390/ma18214857

**Published:** 2025-10-23

**Authors:** Xiaotong Ma, Tianxiang Guo, Yuxiao Xing, Ruize Qin, Huan Long, Chao Bao, Fusheng Cao, Ruixiao Hong

**Affiliations:** 1 School of Civil Engineering, North Minzu University, Yinchuan 750030, China13007929122@163.com (R.H.); 2 School of Civil Engineering and Hydraulic Engineering, Ningxia University, Yinchuan 750030, China; longhuan0803@foxmail.com (H.L.); fushengcao@foxmail.com (F.C.)

**Keywords:** seismic behavior, hybrid structure, energy dissipation capacity, hysteretic curve, digital image processing

## Abstract

Mixed structures with lightweight steel added stories are particularly vulnerable to damage and failure at the joints during seismic events. To evaluate the secondary seismic behavior of the joints in lightweight steel added stories after seismic damage repair, a low-cycle load test was conducted in this study. Following the initial damage, carbon fiber-reinforced polymer (CFRP) was applied for reinforcement, along with epoxy resin for the repair of concrete cracks. The experimental analysis focused on the structural deformation, failure characteristics, and energy dissipation capacity in both the original and repaired joint states. On the basis of the experimental findings, finite element analysis was carried out to examine the influence of varying CFRP layer configurations on the seismic performance of the repaired joints. The results revealed a significant change in the damage pattern of the repaired specimen, shifting from secondary surface damage to significant concrete deterioration localized at the bottom of the column. The failure mechanism was characterized by the CFRP-induced tensile forces acting on the concrete at the column base, following considerable deformation at the beam’s end. When compared to the original joint, the repaired joints exhibited markedly improved performance, with a 33% increase in horizontal ultimate strength and an 85% increase in energy dissipation capacity at failure. Additionally, the rotation angle between the beams and columns was effectively controlled. Joints repaired with two layers of CFRP demonstrated superior performance in contrast to those with a single layer. However, once the repaired joints met the required strength, further increasing the number of CFRP layers had a minimal influence on the mechanical properties of the joints. The proposed CFRP-based seismic retrofit method, which accounts for the strength degradation of concrete in damaged joints due to earthquake-induced damage, has proven to be both feasible and straightforward, offering an easily implementable solution to improve the seismic behavior of structures.

## 1. Introduction

As urban populations continue to grow and the demand to reduce carbon emissions from buildings becomes more urgent, vertical expansion presents a sustainable and innovative solution to address the scarcity of urban space [1,2]. Due to the advantages of having a low weight, allowing for fast construction, and having a low impact on the original building, the use of lightweight steel added stories can effectively address the requirements of building area expansion and altered usage functions throughout the urban renewal process [3]. The transmission of forces between the light steel structure and the reinforced concrete (RC) structure occurs through their connecting joints. Therefore, any sudden changes in stiffness between the upper and lower structures can cause serious damage to the joint of lightweight steel added stories during earthquakes [4,5,6]. Preventing excessive seismic damage to joints and repairing and reinforcing damaged joints can facilitate rapid recovery in the functional use of post-disaster building structures as the health status of the joints determines the overall structural performance. This approach avoids the need for post-disaster demolition and reconstruction [7,8]. Nevertheless, the current seismic code primarily offers recommendations for structures using uniform materials and does not address hybrid structures with vertical irregularity. The distinctive structural configuration of hybrid structures has prompted several studies investigating their overall seismic response. Askouni and Papagiannopoulos [9] analyzed hybrid RC–steel building frames, highlighted the lack of specific seismic guidelines for such structures, and proposed design enhancements based on dynamic tests conducted under severe seismic conditions. Askouni [10] investigated the seismic impact of soil deformability on hybrid RC–steel buildings and proposed enhanced design rules based on comparative dynamic analyses. Katsimpini [11] studied the seismic performance of hybrid concrete–steel structures, identifying shortcomings in current design codes that fail to account for the diverse dynamics of dual-material buildings and the influence of sequential earthquakes and soil–structure interactions on structural responses. Salameh, Shayanfar, and Barkhordari [12] evaluated the seismic response of innovative concrete–steel hybrid wall systems using nonlinear dynamics analyses and derived relations to estimate seismic displacements and other behavioral factors. They revealed the notable impact of the number of stories and uniformity on the structural reaction outcomes. Ghanbari et al. [13] performed double incremental dynamic analysis on an RC–steel hybrid frame, plotted its fragility curves, and demonstrated it to have a greater collapse resistance and a 33% lower collapse probability than a purely RC frame. Zhang [14] proposed a stochastic homotropy method to analyze vertically irregular structures by modeling elastic moduli as random fields and obtaining stable natural frequencies and modal shapes. Validation on concrete–steel structures revealed this model to largely outperform traditional ones. Kiani [15,16,17] explored the seismic resilience of hybrid buildings focusing on the optimal placement of the transitional story between different structural systems. Through incremental dynamic analysis, they observed that positioning the transitional story at one-third of the building’s height from the top significantly reduces seismic vulnerability across various configurations. Papagiannopoulos [18] outlined two approaches to calculate modal damping ratios in hybrid RC–steel buildings. Both methods effectively predict the linear seismic responses of a five-story structure using numerical simulations, with the choice of method depending largely on software capabilities.

After determining the seismic response characteristics of hybrid structures, it is essential to achieve load transfer and a reliable connection between reinforced concrete structures and steel structures for the engineering application of mixed structures. However, the current research on mixed structures mainly focuses on the overall structural response, and there is relatively little research on the connection joints between reinforced concrete and steel structures. Pan [4,19] proposed a novel outsourcing joint and a reinforced joint; both feature higher load-bearing capacity, greater stiffness, and superior seismic performance for mixed structures. Regarding the sudden variations in stiffness and force transfer mechanisms, Bahri [5,20] explored the seismic responses of height-varied buildings using numerical and experimental methods, and they proposed a novel column splice connecting concrete and steel sections by testing its performance under cyclic loads to evaluate deform ability and energy dissipation. Gao [6] examined the seismic resilience of a novel connection joint for mixed structures through cyclic loading tests; they found that variations in the size of the steel column and the ratio of axial force could enhance joint strength and energy dissipation, and they provided design recommendations for optimizing these joints in mixed buildings. An effective joint must not only fulfill diverse functional requirements and withstand load impacts but also support efficient post-disaster repair and reconstruction [21,22]. Helal et al. [23] established metal straps to repair and reinforce external RC beam–column joints, significantly increasing their shear capacity increased by as much as 42% compared to their unprotected counterparts. Lu et al. [24,25] studied the ability to resist seismic forces and the performance in post-earthquake recovery of a self-centering frictional beam–column connection, which showed obvious advantages in terms of total residual deformation, seismic collapse resistance, immediate occupancy, and reparability. Hung et al. [26,27] explored the effectiveness of ultra-high-performance concrete jackets reinforced with high-strength steel mesh in restoring seismically damaged exterior beam–column joints, offering a unique rehabilitation technique. Zhang et al. [28] introduced an innovative device for repairing earthquake-damaged unbonded post-tensioned hybrid connections, with the potential to fulfill energy dissipation and stiffness requirements for different damage levels. Zhang et al. [29] created beam–column joints made of high-strength recycled aggregate concrete, reinforced with ultra-high-strength steel bars and plates, demonstrating improved bearing and deformation capacities post-rehabilitation, particularly for steel-tube encased columns. Nguyen and Tan [30] explored the progressive collapse resistance of precast concrete joints reinforced with headed bars after slight and moderate earthquake damage. They found that the damaged joints retained some collapse resistance through flexural and catenary actions despite decreased stiffness and deformation capacity. Ren et al. [31] examined the seismic enhancement of post-earthquake-damaged reinforced concrete frames using engineered cementitious composites, and they established engineered cementitious composites as an effective repair material for earthquake-damaged structures.

Although earlier studies have validated the effectiveness of the earthquake-damaged joint repair method in improving the seismic behavior of structures, it is worth noting that no research has investigated its specific application in enhancing the behavior of joints in mixed RC–steel frames. In this study, CFRP materials were employed for the repair of earthquake-damaged joints, and quasi-static tests and finite element analysis were performed on the repaired joints. The test phenomena, mechanical properties, and ductility of the original joints were compared to those of the repaired joints.

## 2. Specimen Preparation and Test Methods

### 2.1. Specimen Preparation

**Figure 1:**
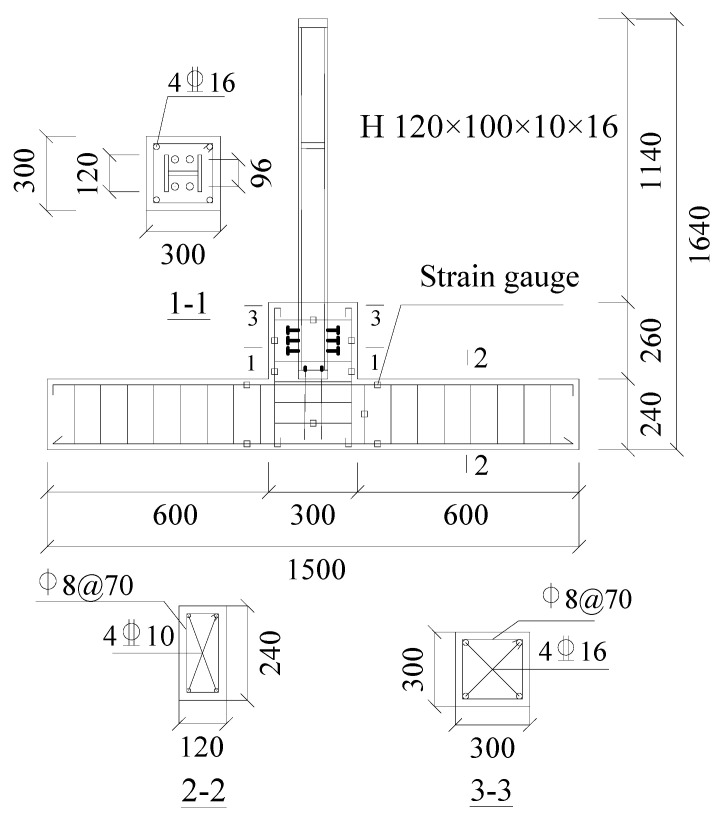
Diagrammatic representation of the test specimen and its parameters (mm).

For the practical engineering implementation of adding stories using lightweight steel, we initially poured 120 mm × 240 mm concrete beams and 300 mm × 300 mm concrete columns for the lower stories. The base materials used in this study were C30 grade concrete and Q235B steel. After 28 d of standard curing the base concrete beam and column, we drilled the positioning hole, cleared the debris within, and applied the rebar planting adhesive. A bolt was then thoroughly blended with the adhesive by using the electric tool, and C50 non-shrinkage concrete was used for compact leveling after solidification. Subsequently, the lightweight steel added stories were installed by welding six M14 bolts onto the concrete base of the H-shaped steel column to enhance the contact area (Figure 1).

**Figure 2:**
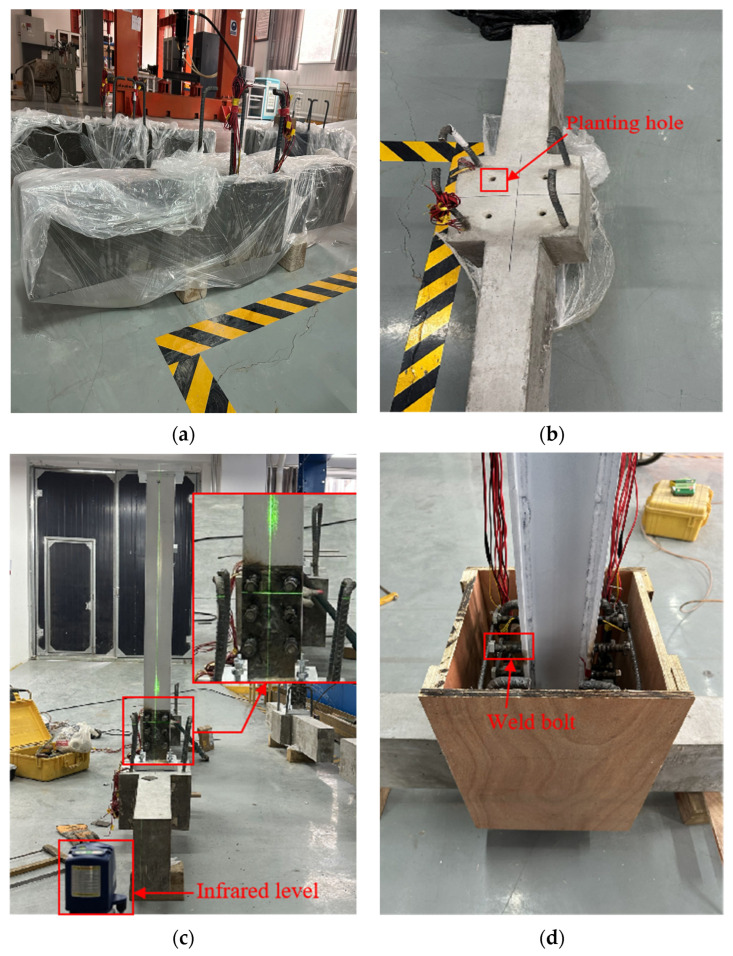
Test specimen construction steps. (**a**) Substructure; (**b**) Post-installed rebars; (**c**) Structural leveling; (**d**) Secondary pouring.

In this study, the stress values were determined through uniaxial tensile testing. The average ultimate tensile strengths obtained from these tests were 487.56 MPa for the stirrup, 587.14 MPa for the longitudinal reinforcement of the beam, and 614.58 MPa for the longitudinal reinforcement of the column. The stress was calculated based on the force applied during the test and the cross-sectional area of a particular part of the considered region of the element. During the construction of the test specimen, the horizontal and vertical installation of the H-shaped steel column was the key factor in ensuring the success of the test [32,33,34]. The infrared level was employed for precise leveling, and the construction process of the test specimen is illustrated in Figure 2.

### 2.2. Test Methods

**Figure 3:**
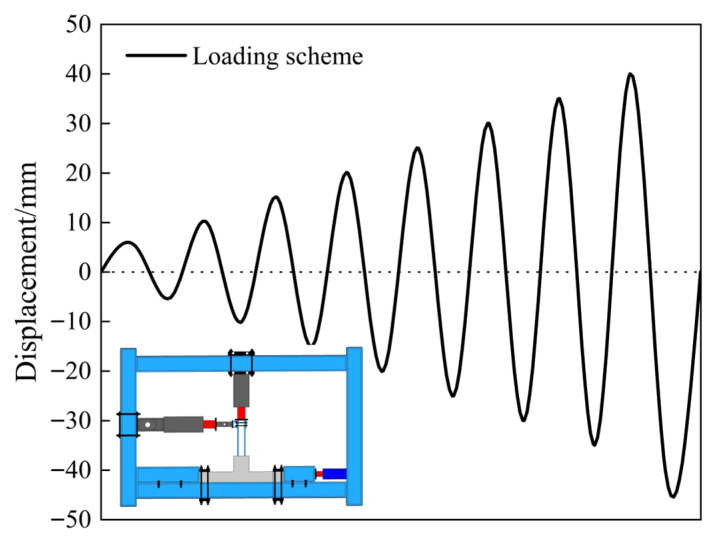
Loading mechanism and scheme.

The experiments were carried out using Structural Engineering Innovation Testing Platform. Specifically, the vertical servo-hydraulic actuator had a capacity of 500 kN, and the horizontal servo-hydraulic actuator had a capacity of 300 kN. During the tests, digital image correlation (DIC) was employed as the optical measurement technique to comprehensively monitor the deformation of the specimens. Throughout the experiment, the vertical load was carefully controlled within a stability tolerance of ±1% around the target value to minimize measurement errors. The pseudo-quasi-static test method was employed to control the top displacement of the H-shaped steel column [35,36,37,38]. The upper load was simulated, and a constant axial force was applied vertically with an axial compression ratio of 0.15. A servo-hydraulic press was used to maintain the dynamic balance throughout the loading process. Horizontal loading began when the load on the upper part of the column reached the intended axial force; the horizontal load was applied to the top of the column according to the loading mechanism and scheme shown in Figure 3.

### 2.3. Reinforcement and Repair

**Figure 4:**
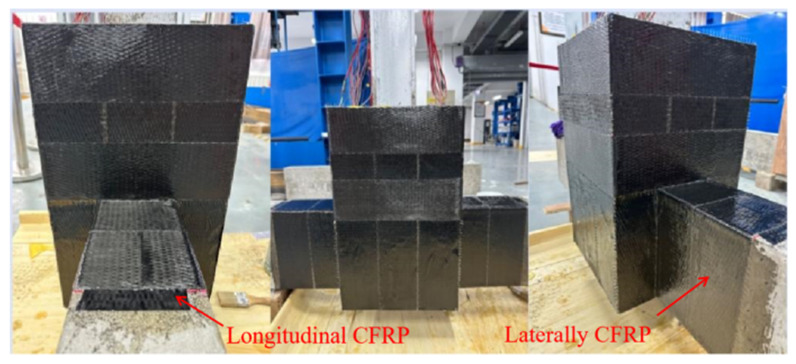
Diagram of repaired joint.

After polishing the surface of the original joint (Figure 4), the damaged area was repaired with epoxy resin. Specifically, the epoxy resin and hardener were mixed at a weight ratio of 2:1 and thoroughly stirred at room temperature. The resulting adhesive mixture was then applied to the concrete surface. Subsequently, carbon fiber fabric (CFRP), with a width of 100 mm and a thickness of 0.167 mm, was bonded to the surface in a crisscross pattern to provide reinforcement.

Following the application of the CFRP layers, the repaired structure was cured at room temperature for 72 h. This curing period was implemented to ensure adequate bonding and complete hardening of the epoxy resin prior to the initiation of loading. After the curing process was completed, the structure was subjected to testing using the same loading system as employed in the previous tests.

## 3. Test Results and Evaluation

### 3.1. Test Observation

**Figure 5:**
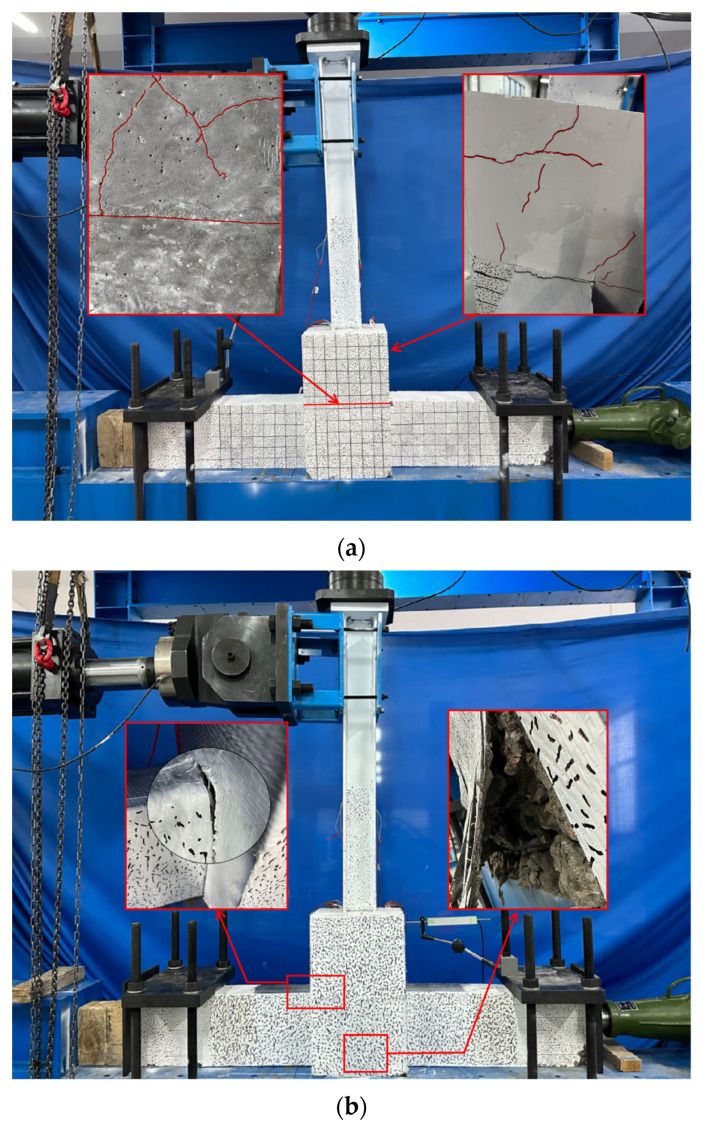
Failure mode. (**a**) Original joint; (**b**) CFRP-reinforced joints.

Following the loading tests, cracks in the original joints were found to be mainly concentrated on the secondary casting surface (Figure 5), with mixed smaller cracks and a large crack on the secondary pouring surface. The specimen was no longer suitable to bear continued loading, and hence, the test was stopped. In the case of the CFRP-reinforced joint, the fibers at the beam ends were initially subjected to tension, causing the concrete to gather at the beam’s end. This led to significant plastic deformation. Eventually, the CFRP fibers pulled on the concrete at the column base, leading to structural damage.

### 3.2. Hysteresis Curve

**Figure 6:**
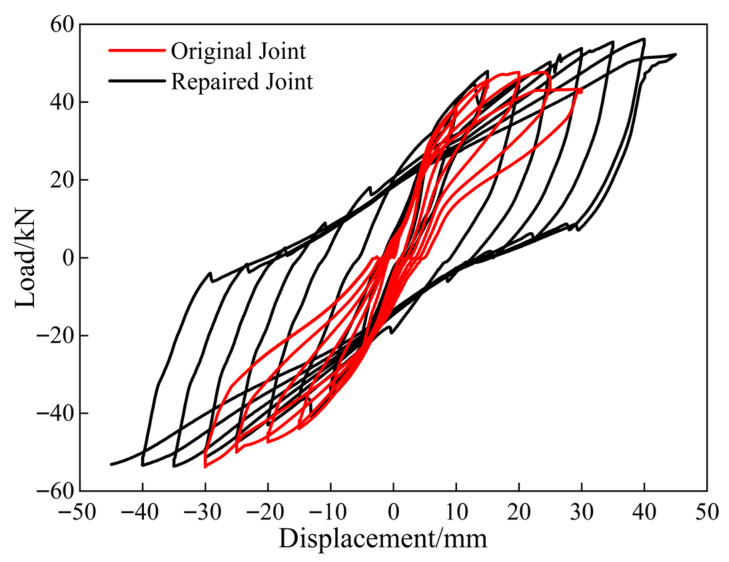
Hysteresis curve.

Figure 6 shows a comparison of the hysteresis curves of the original and repaired joints under low-cycle reciprocating loading. The hysteresis loops of the two types of joints were highly consistent for a horizontal displacement of ≤10 mm, with a small, linear residual deformation. As the horizontal displacement reached 45 mm, the bearing capacity of the repaired joint began decreasing. Compared to the original joints, the repaired joints initially showed a lower horizontal bearing capacity, which then increased, with the difference being more pronounced under reverse load application. This effect was primarily due to the cumulative damage between CFRP and concrete causing the peeling of concrete during forward load application. The hysteresis curves revealed the repaired joints to have stronger horizontal bearing capacity and higher horizontal displacement limit with CFRP reinforcement.

**Figure 7:**
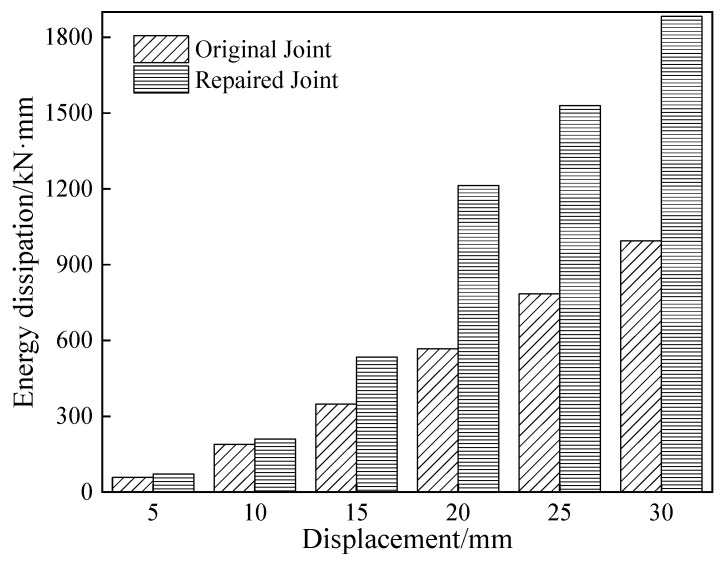
Energy dissipation.

The specimens continuously absorbed energy under low-cycle reciprocating loads; the area of the closed curve under a given level of displacement was obtained as the energy loop (Figure 7).

The energy dissipation capacity at various displacement levels was used to assess the seismic behavior of the specimen. The variations in loading displacement were measured relative to the energy enclosed by the loading circle. At the initial stages of loading, both types of joints remained in the elastic phase, exhibiting low energy consumption, gradual growth rates, and no obvious difference in energy dissipation. As displacement increased, the energy dissipation capacity of the repaired joint increased sharply. It exhibited an energy ring with a radius/diameter of 20 mm, twice that of the original joint. The hysteresis loop area of CFRP-reinforced joints, under each displacement load, was larger than that of the original joints. With the total hysteresis loop area of the former being 85% larger than that of the latter. The repaired joints had a higher energy dissipation capacity and exhibited better ductility characteristics. These results validated the reliability of CFRP-reinforced damaged joints after earthquakes.

### 3.3. Skeleton Curve

**Figure 8:**
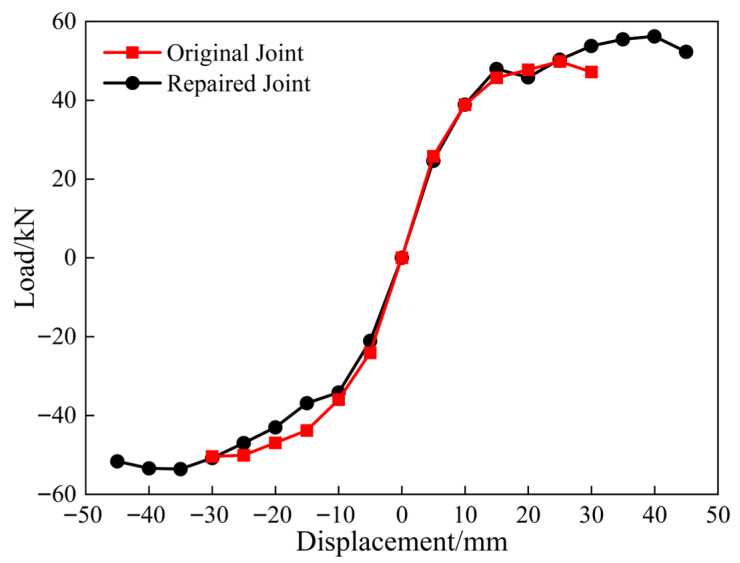
Skeleton curve.

The peak load values corresponding to various displacement levels during the cyclic loading process were extracted and sequentially connected to obtain the load skeleton curve for the top loading point of the column (Figure 8). The skeleton curves of the repaired and original joints exhibited similar trends. In the elastic stage, the curve slope was steep, and the load capacity increased significantly. After the specimen reached yielding, the curves exhibited an S-shaped profile, with a rapid decrease in slope and a gradual increase in load capacity. The horizontal bearing capacity of the CFRP-reinforced joint was completely repaired compared with the original joint, exhibiting characteristics similar to those of the building before damage. In terms of concrete damage, a strong push–weak pull phenomenon was observed. The skeleton curve indicated that the repaired joint had a higher peak horizontal displacement and improved ductility compared to the original joint.

### 3.4. Stiffness Degradation Curve

**Figure 9:**
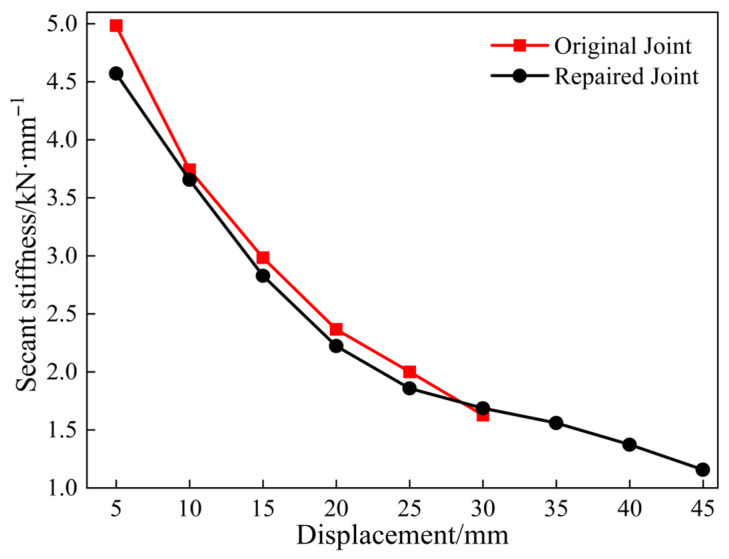
Stiffness degradation curves.

The stiffness degradation curve is used to evaluate the extent of damage and assess the structural health, effectively reflecting the fatigue experienced under low-cycle loading. Therefore, the secant stiffness of the joints [19] was measured to reflect the stiffness degradation degree with respect to the loading displacement (Figure 9). No distinct inflection point was observed in the stiffness degradation curves of either type of joint, both exhibiting an initial rapid decline followed by a more gradual reduction. The whole stiffness of the repaired joint was found to be lower than that of the original joint, with an initial stiffness difference of 8%. For a horizontal loading displacement of 30 mm, the stiffness of the original and repaired joints degenerated to 32.6% and 36.8% of their initial values, respectively. Although the total stiffness of the repaired joint remained slightly lower than that of the original joint, it exhibited a more gradual stiffness degradation process and demonstrated the added benefit of a delayed reduction in bearing capacity.

## 4. Detection and Analysis Based on Digital Image Correlation Techniques

Digital image correlation (DIC) is a non-contact, high-precision detection technology that enables full-field measurement and real-time monitoring [39,40,41]. Images of the specimen are captured using a high-speed camera, with marker points in different images compared to calculate the displacement and deformation of the target area. This process can effectively help us understand the mechanical properties, deformation behavior, and failure mechanism of a given structure under load testing. It provides a scientific foundation for the damage accumulation, optimal design, and safety assessment of structures.

### 4.1. Shear Deformation

**Figure 10:**
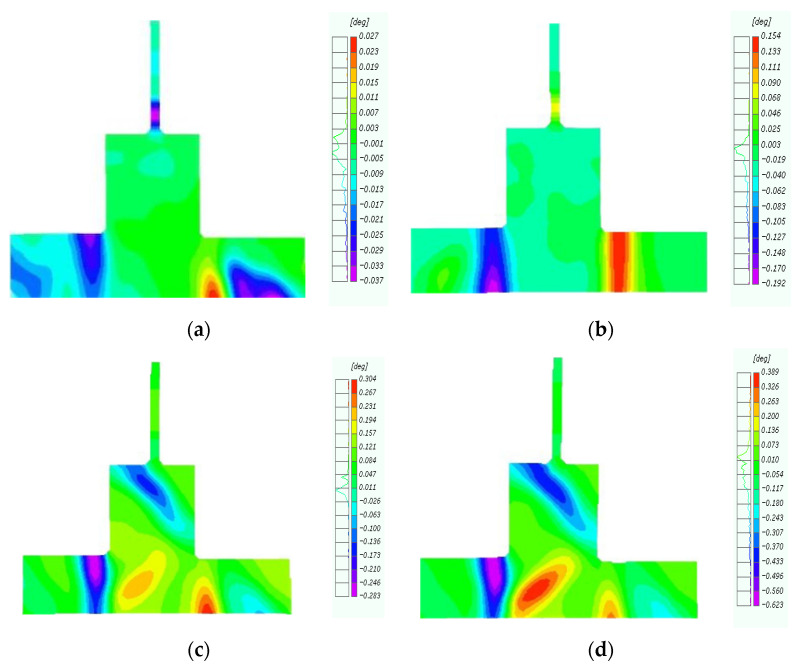
Shearing angle of original joint. (**a**) Displacement of 5 mm; (**b**) Displacement of 10 mm; (**c**) Displacement of 20 mm; (**d**) Displacement of 30 mm.

During earthquakes, relative displacement or sliding occurs within the concrete interior, causing plastic deformation and irreversible shear damage. It is of great significance to shear the deformation behavior and mechanical properties of specimens by shear deformation. Therefore, we further examined the shear deformation behavior of the original and repaired joints in the forward loading process via digital image processing to intuitively analyze the failure characteristics of the specimens (Figure 10). The concrete shear deformation contour for the original joints revealed the initial position of shear deformation to be at the top of the left-end beam and the bottom of the right-end beam (Figure 10a). When the horizontal displacement reached 10 mm, the shear deformation was concentrated at the extremity of the concrete beam (Figure 10b). As the displacement increased, concrete deformation occurred at the outer column base at a 45° angle at 20 mm (Figure 10c). Finally, at 30 mm, the concrete column deformed symmetrically about the secondary pouring surface. The loading test was stopped at this stage due to the appearance of large cracks in the concrete.

**Figure 11:**
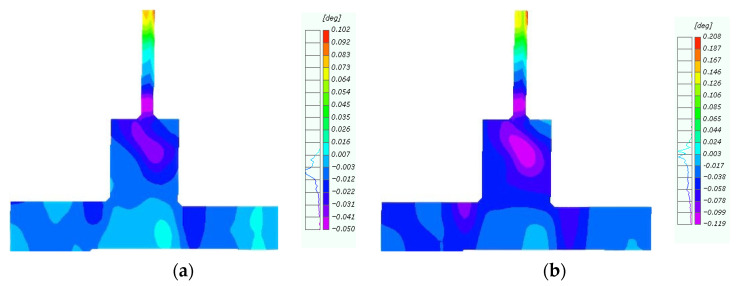

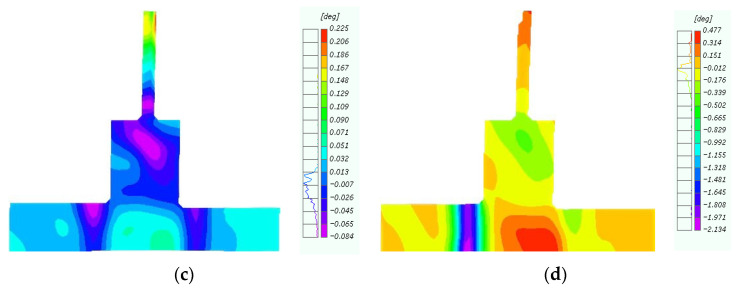
Shearing angle of repaired joint. (**a**) Displacement of 5 mm; (**b**) Displacement of 15 mm; (**c**) Displacement of 30 mm; (**d**) Displacement of 45 mm.

The CFRP-reinforced joint exhibited multi-point shear deformation, primarily along a 45°angle at the concrete-wrapped column base, the bottom of the column, and the edge of the CFRP (Figure 11a,b). CFRP improved the integrity of the joint core, and the repaired joint exhibited clear shear deformation at the interface between the H-section steel and the outer base of the column (Figure 11c). Similar to the loading tests, the experiment was terminated when the CFRP caused the concrete at the bottom of the column to peel off (Figure 11d).

Overall, the shear deformation behavior of (i) the original joint was relatively concentrated, with a clear trend, and (ii) repaired joints exhibited a multi-point trigger, mainly concentrated in the middle column and beam end. Compared to the original joint, the repaired joint demonstrated enhanced integrity, avoiding the occurrence of large cracks and deformations.

### 4.2. Damage Analysis

**Figure 12:**
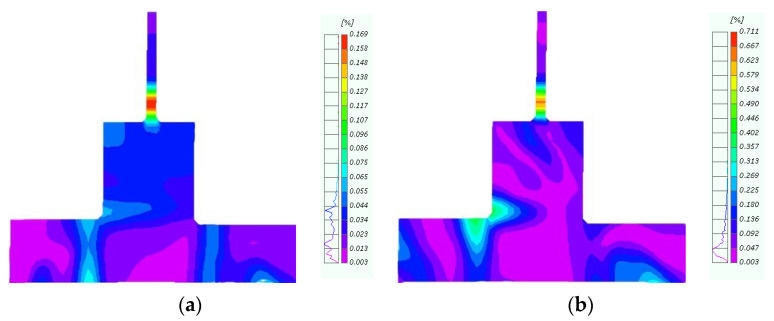

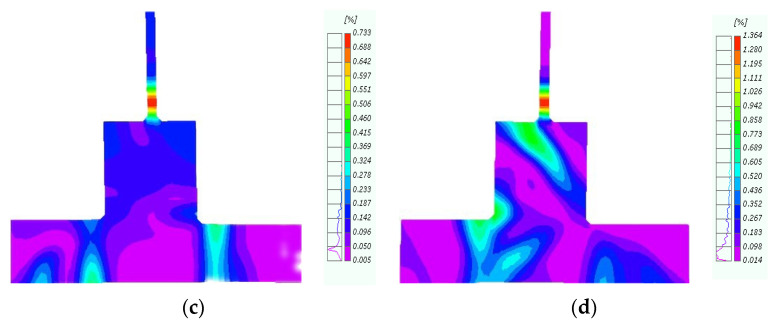
Failure mode of original joint damage process. (**a**) Initial state; (**b**) Development stage; (**c**) Extension stage; (**d**) Failure stage.

Under low-cycle reciprocating loads, damage to concrete structures accumulated, and cracks continued to extend. The structural failure characteristics of the specimen were analyzed through strain measurements and damage assessment on the concrete. The concrete damage areas in the original joint were centered at the pouring joint (Figure 12a), extending from the interface until plastic deformation occurred at a 45°angle at the outer column base of the concrete (Figure 12b), clearly highlighting the weak sections (Figure 12c). Particularly, the H-shaped steel experienced significant stress concentration at the connection joints (Figure 12d).

**Figure 13:**
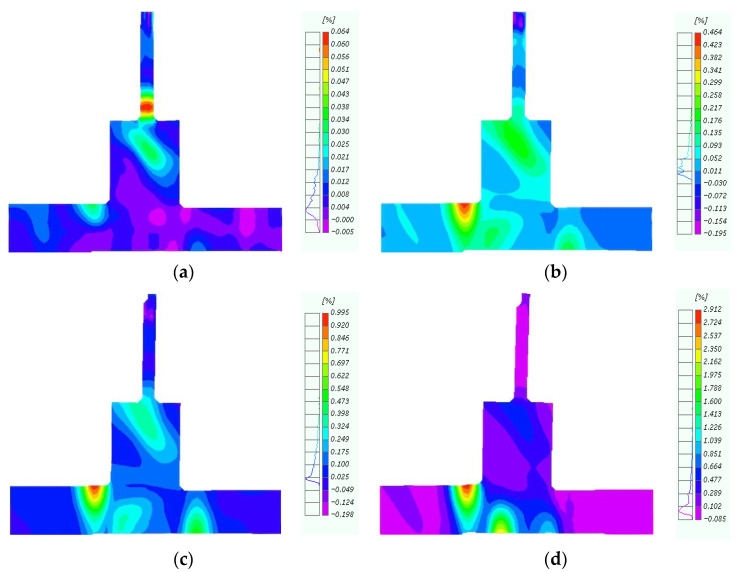
Failure mode of repaired joint damage process. (**a**) Initial state; (**b**) Development stage; (**c**) Extension stage; (**d**) Failure stage.

The repaired joint initially exhibited a 45° inclined crack originating from the upper end of the column, which progressively widened and extended with increasing horizontal displacement (Figure 13a). This crack formation was attributed to the significant tensile stress concentrated at the beam end (Figure 13b). As loading continued, the CFRP reinforcement at the beam end effectively increased its stiffness and tensile capacity, which, while preventing further local damage at the beam end (Figure 13c), also caused a redistribution of forces within the joint (Figure 13d).

As the beam end became stiffer, the internal forces were redirected towards the column base, where the stress concentration increased. Once the concrete at the beam end began to degrade, the H-shaped steel section transferred more of the load to the concrete column base, leading to an integrated structural response between the steel column and the concrete base. This interaction intensified the compressive and shear forces at the base of the column, which ultimately caused concrete rupture.

Therefore, the above analysis indicates that the damage to the concrete had minimal impact on the reinforcing effect of the CFRP. Moreover, the damage locations of the repaired joint were different from those of the original joint, proving the reliability of the method.

### 4.3. Angle Between Beam and Column

**Figure 14:**
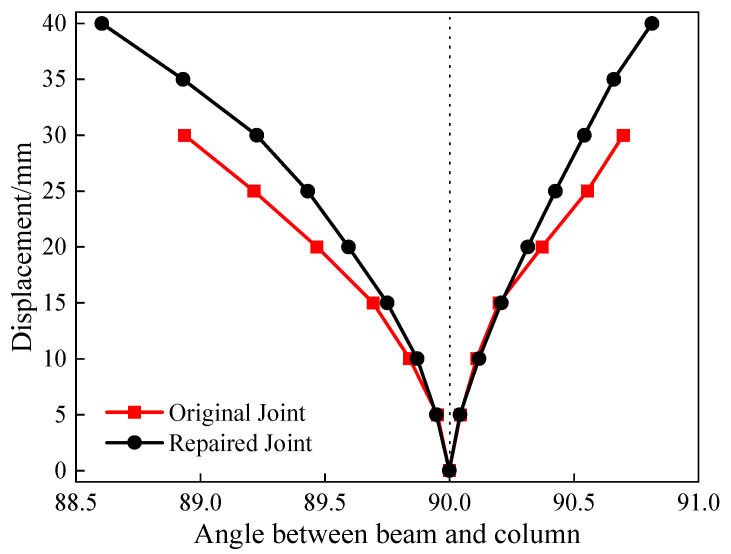
Diagram of angle variation.

The vertical mass and stiffness of the lightweight steel added stories undergo abrupt changes at the connection joints, which are prone to significant deformation and displacement during earthquakes. Therefore, effectively controlling the angle between the beam and column in the repaired joints of the added stories is critically important [42]. The variations in the angle between the beam and column with horizontal displacement for the original and repaired joints almost coincided during the initial stage (Figure 14). However, the trends began to diverge at a forward displacement of 15 mm and a reverse displacement of 10 mm. From the relative point of view of the beam and column, the CFRP effectively restrained the damage to the concrete in the repaired joints, substantially improving structural integrity.

### 4.4. Concrete Strain

**Figure 15:**
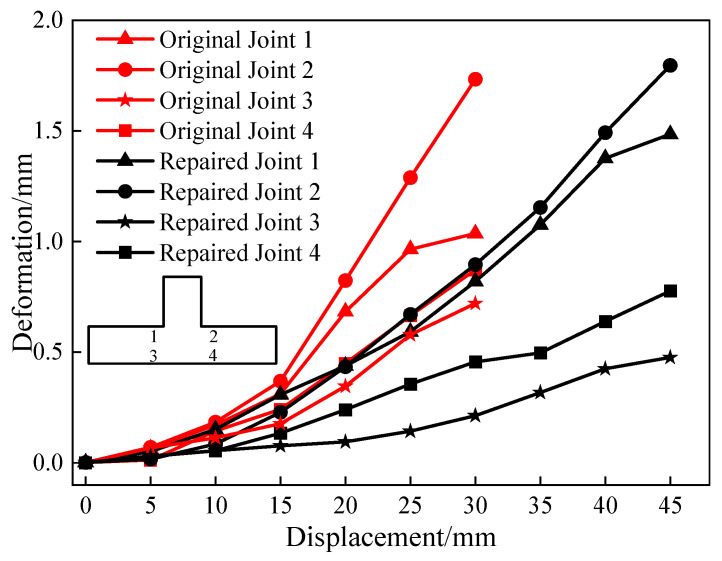
Concrete deformation.

The strain values of the concrete beam under various peak displacements during the forward loading process were finally obtained (Figure 15). For the beam, the strain decreased (i) from top to bottom and (ii) from right to left. The concrete strain at the beam’s end section was smaller for the CFRP-reinforced joints compared to the original joints, indicating a significant protective effect on the joint core. These results verified the feasibility of repairing damaged hybrid structure joints with CFRP.

## 5. Numerical Simulation Analysis

To further investigate the factors influencing the performance of CFRP-reinforced joint, finite element analysis was conducted based on experimental results. This analysis aimed to explore the impact of different CFRP layer configurations on the seismic performance of repaired joints, providing significant insights for the design and construction of such structures.

### 5.1. Validation of the Finite Element Model

The finite element model was developed using the same geometric dimensions, loading conditions, and constraints as those in the experimental tests to validate the accuracy of the finite element analysis. The results of the finite element analysis were compared with the test results of ordinary concrete joints, including the skeleton curve, stiffness degradation curve, and failure modes.

**Figure 16:**
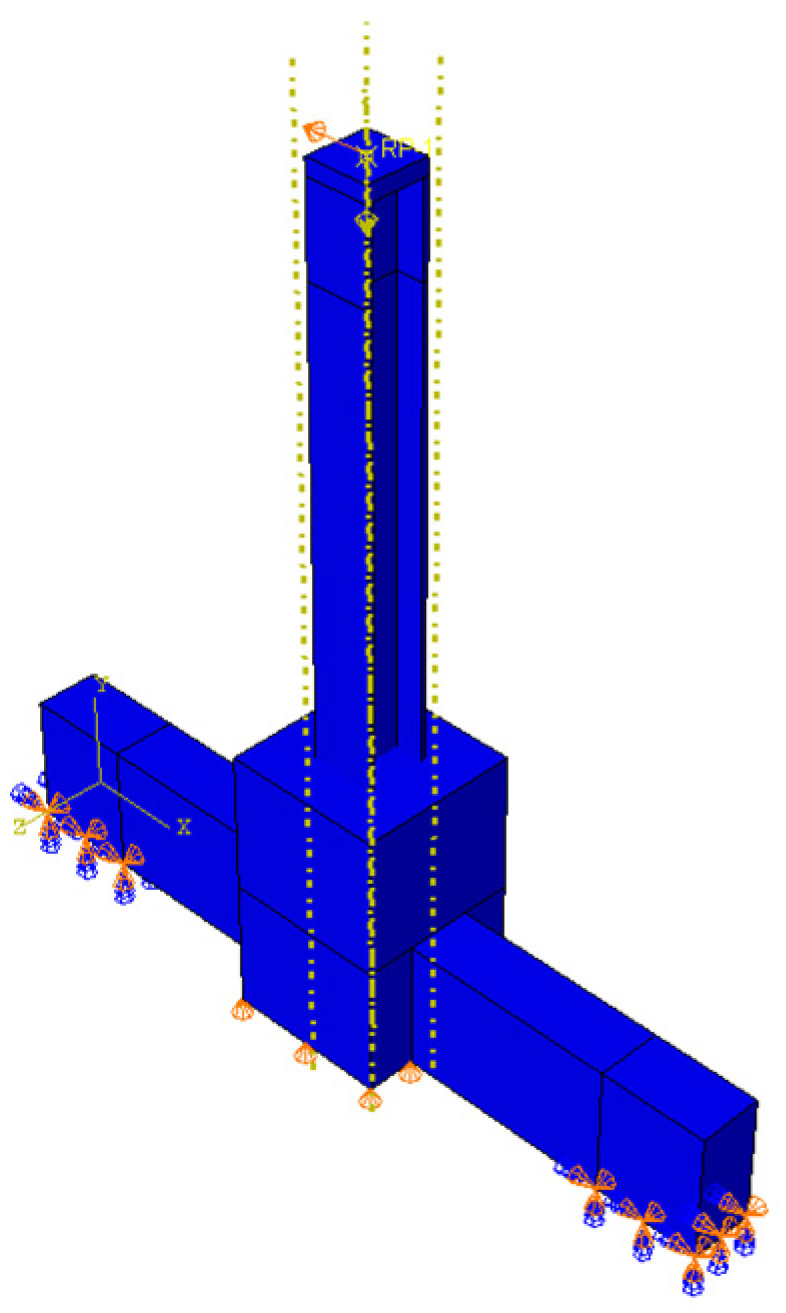
Finite element model.

Numerical simulations were conducted using the finite element software ABAQUS (2017). The concrete beams and columns, H-shaped steel plates, and outer column feet were defined using the 8-node hexahedral linear reduced integration solid element C3D8R. The reinforcement framework was modeled using the 2-node linear three-dimensional truss element T3D2 [43,44]. The finite element model is presented in Figure 16. This model has 2238 nodes and 1446 elements. For the concrete materials, the CDP plastic damage model available in ABAQUS was utilized to evaluate the mechanical properties, accounting for cracking damage due to both tension and compression. The damage process was considered continuous, with compressive cracks not recovering during stress state transitions. The default stiffness recovery factor in ABAQUS was applied and the constitutive models for the rebar and steel adhered to the maximum Huber–von Mises yield criterion and the corresponding flow criterion [45]. Additionally, the rebar parameters were adjusted to reflect the engineering characteristics of light steel layer reconstruction in existing buildings, as detailed in Table 1.

**Figure 17:**
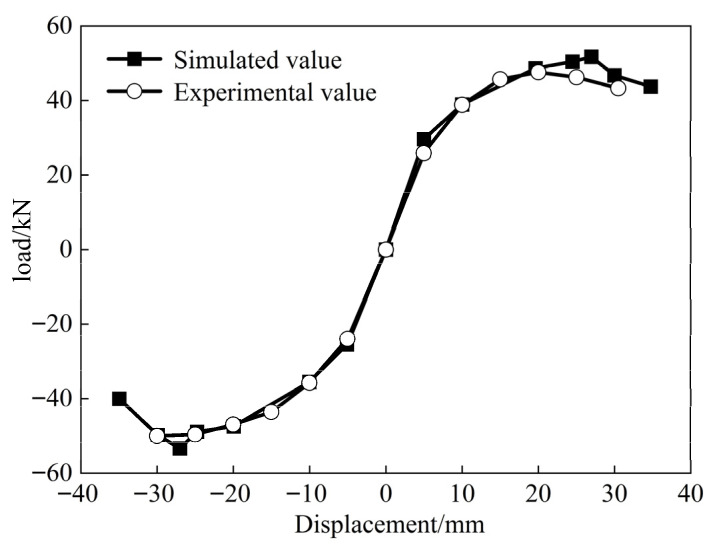
Skeleton curve.

The peak load values corresponding to the first cyclic loading at various displacement levels were extracted for both the ordinary concrete joints and the finite element model. The displacement-load skeleton curves of the column top loading points were then sequentially connected. As shown in Figure 17, the skeleton curves obtained from the finite element model and the experimental results exhibited similar overall trends, with the numerical model predicting slightly higher values. This discrepancy can be attributed to the non-ideal conditions of the test, where external factors resulted in a more rapid reduction in bearing capacity compared to the predictions of the finite element model. However, the difference in horizontal displacement loading was only 3 mm. Regarding the horizontal load values at key points, the maximum discrepancy between the test and finite element results, excluding the drop point, was 8.3%. The difference in horizontal bearing capacity at the drop point was only 5.6%, with the error in the load characteristic values for both being less than 10%.

**Figure 18:**
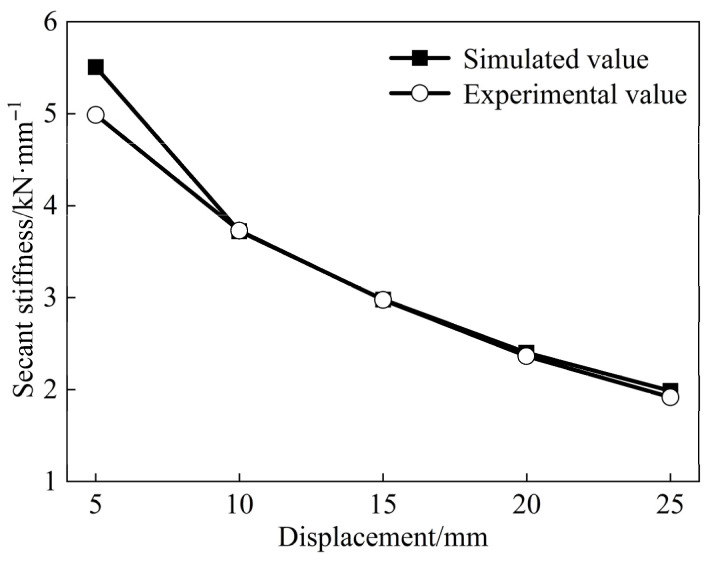
Stiffness degradation curve.

The stiffness degradation curve effectively predicts the response characteristics under earthquake loading and reflects the seismic performance of the joints. The stiffness degradation curve of the finite element model closely matched the experimental results (Figure 18). Although the finite element model initially exhibited higher stiffness, the change rates of both results between 10–25 mm of loading displacement were 12.0% and 11.5%, respectively, with a difference of only 0.5%. The degradation trends of both curves were nearly identical, and their patterns of change were consistent. This confirms the exactness of the finite element model presented in this study, providing strong validation for its use in subsequent research.

### 5.2. Influence Analysis of the Number of CFRP Layers

CFRP material is extensively utilized in practical engineering owing to its advantages of lightweight, high strength, corrosion resistance, and durability. It is particularly beneficial for projects with limited construction space and complex structural configurations, as it can be tailored to the structure’s shape to improve stiffness, seismic performance, and load-bearing capacity. In engineering practice, balancing cost and performance is a key consideration when determining reinforcement strategies.

**Figure 19:**
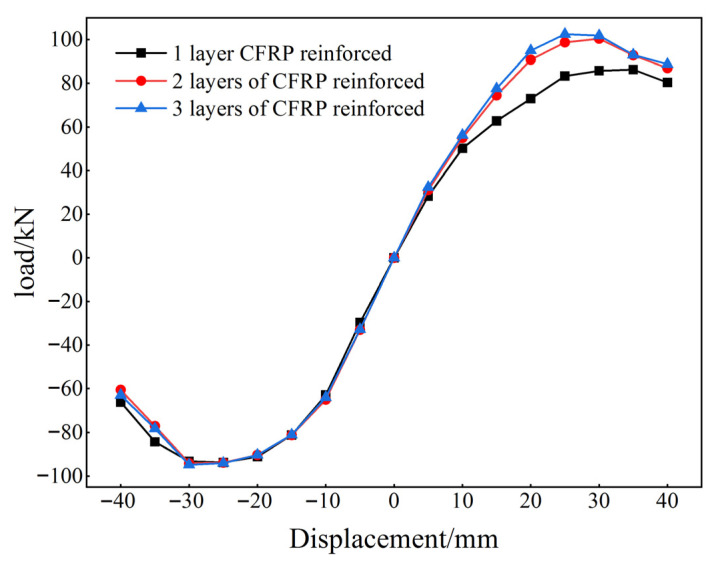
Skeleton curve.

Figure 19 shows the skeleton curves for joints reinforced with one, two, and three layers of CFRP. The graph illustrates that the horizontal displacements of joints with different CFRP layer configurations exhibit similar declines in bearing capacity once the horizontal displacements reach 30 mm. When the number of CFRP layers was increased to two, the joint bearing capacity improved significantly after the horizontal displacement exceeded 10 mm, with a 20% increase in bearing capacity at a displacement of 20 mm. The bearing capacity of the three-layer CFRP was almost identical to that of the two-layer configuration. As some reinforcement layers reached their yield stress during forward loading, the lateral load capacity during reverse loading was lower than that during forward loading. Additionally, the protective effect of the CFRP on concrete cracks and structural damage was similar across configurations, indicating that the number of CFRP layers had minimal impact on the horizontal load capacity of the structure. The analysis indicates that applying two layers of CFRP at the joints provides an optimal solution for meeting structural performance requirements while effectively minimizing the impact of construction errors on structural performance in practical engineering.

**Figure 20:**
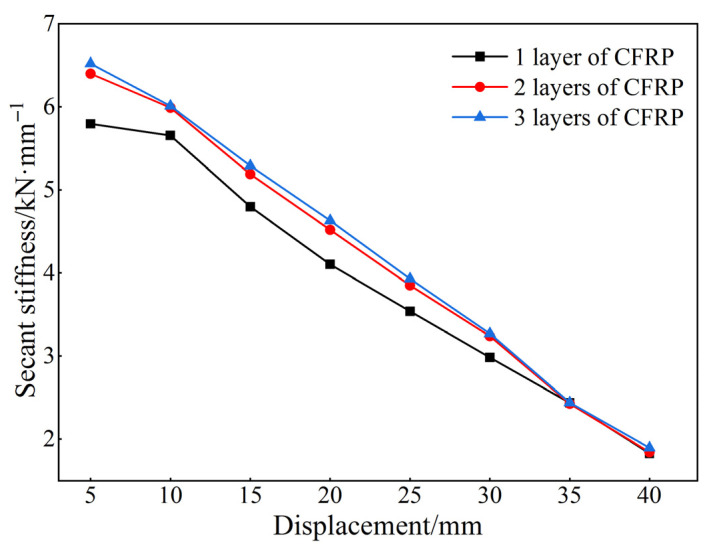
Stiffness degradation curve.

The number of CFRP layers applied to a joint also plays a critical role in determining the seismic behavior of the retrofitted structure. As depicted in Figure 20, an analysis of stiffness degradation curves for different numbers of CFRP layers reveals that the CFRP begin to tear and partially rupture when the horizontal displacement reaches 35 mm. The joint stiffness with a single layer of CFRP is significantly lower than that with two or three layers, while the stiffness of joints with two or three layers is nearly identical. These results indicate that while additional CFRP layers lead to an increase in peak load capacity, the incremental improvement in stiffness becomes less significant beyond the first layer. Therefore, the effect of increasing CFRP layers on stiffness appears to diminish after initial strengthening.

### 5.3. Evaluation of the Effect of the CFRP Layers on Concrete Damage

**Figure 21:**
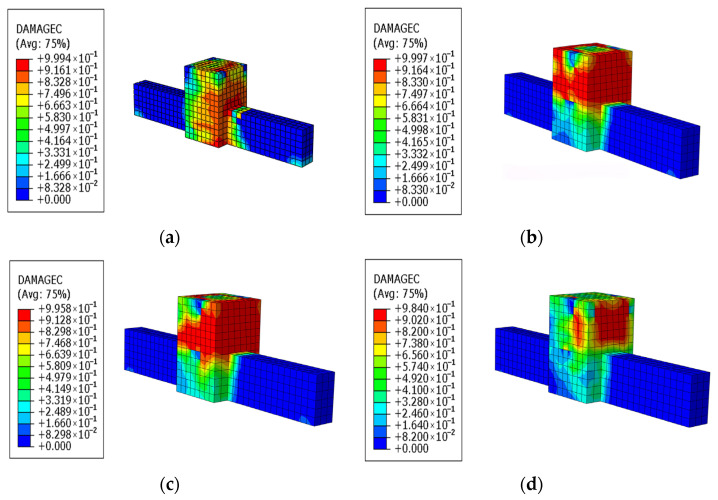
Concrete Damage Diagram. (**a**) Without CFRP fabric; (**b**) 1 layer of CFRP fabric; (**c**) 2 layers of CFRP fabric; (**d**) 3 layers of CFRP fabric.

By comparing the concrete damage in the joint core area with and without CFRP, as well as under different CFRP layer configurations in Figure 21, it is evident that the entire concrete in the joint core area was damaged when no CFRP was applied. The application of CFRP significantly reduced the concrete damage within the joint core area, with the overall damage shifting from complete damage to partial damage at the column footing outside the joint core area. Increasing the number of CFRP layers further improved the concrete condition at the outsourced column footing, although the extent of improvement became less pronounced with additional layers. This can be observed by comparing the damage across different CFRP layer configurations.

## 6. Conclusions

This study into the repair of post-earthquake damage in mixed reinforced concrete–steel frame structures using CFRP led to the following conclusions:

(1) The seismic capacity of the damaged mixed reinforced concrete–steel frame structure was significantly enhanced through CFRP retrofitting. After retrofitting, all mechanical properties of the repaired joints, except for stiffness, showed improvement compared to the original joints. Specifically, compared with their pre-retrofitting state, the CFRP-reinforced joints exhibited increased ductility and enhanced energy dissipation capacity.
(2) For the mixed reinforced concrete–steel frame structure with severely damaged joints subjected to reciprocal loading, CFRP retrofitting effectively restored and improved the seismic capacity of the joints.
(3) The proposed CFRP seismic retrofit design method, which accounts for the strength degradation of concrete in damaged joints due to earthquake-induced damage, has proven to be feasible, straightforward, and easily implementable.
(4) When joints are reinforced with both vertical and horizontal CFRP layers, the lateral load-bearing capacity increases significantly when two CFRP layers are used compared to one. However, once the CFRP reinforcement meets the required strength, further increases in the number of layers yield only marginal improvements in the lateral load-bearing capacity of the joints and the concrete protection effect.

## Figures and Tables

**Table 1:** The rebar parameters.

No.	Elasticity Modulus (*E*_s_/MPa)	Yield Strength (*f*_y_/MPa)	Ultimate Strength (*f*_u_/MPa)
A8	2.1 × 10^5^	299.30	440.19
C12	2.0 × 10^5^	335.64	468.71
C16	2.0 × 10^5^	356.51	492.20

## Data Availability

The original contributions presented in this study are included in the article. Further inquiries can be directed to the corresponding authors.
